# Protective Immunity in Mice Immunized With *P. vivax* MSP1_19_-Based Formulations and Challenged With *P. berghei* Expressing *Pv*MSP1_19_

**DOI:** 10.3389/fimmu.2020.00028

**Published:** 2020-02-19

**Authors:** Irina Dobrescu, Tarsila Mendes de Camargo, Alba Marina Gimenez, Oscar Murillo, Kelly Nazaré da Silva Amorim, Claudio Romero Farias Marinho, Irene Silva Soares, Silvia Beatriz Boscardin, Daniel Youssef Bargieri

**Affiliations:** ^1^Department of Parasitology, Institute of Biomedical Sciences, University of São Paulo, São Paulo, Brazil; ^2^Department of Clinical and Toxicological Analyses, School of Pharmaceutical Sciences, University of São Paulo, São Paulo, Brazil

**Keywords:** *Plasmodium vivax*, *Plasmodium berghei*, vaccines, MSP1_19_, mouse model

## Abstract

The lack of continuous *in vitro* cultures has been an obstacle delaying pre-clinical testing of *Plasmodium vivax* vaccine formulations based on known antigens. In this study, we generated a model to test available formulations based on the *P. vivax* MSP1_19_ antigen. The *Plasmodium berghei* strains ANKA and NK65 were modified to express *Pv*MSP1_19_ instead of the endogenous *Pb*MSP1_19_. The hybrid parasites were used to challenge C57BL/6 or BALB/c mice immunized with *Pv*MSP1_19_-based vaccine formulations. The *Pv*MSP1_19_ was correctly expressed in the *P. berghei* hybrid mutant lines as confirmed by immunofluorescence using anti-*Pv*MSP1_19_ monoclonal antibodies and by Western blot. Replacement of the *Pb*MSP1_19_ by the *Pv*MSP1_19_ had no impact on asexual growth *in vivo*. High titers of specific antibodies to *Pv*MSP1_19_ were not sufficient to control initial parasitemia in the immunized mice, but late parasitemia control and a balanced inflammatory process protected these mice from dying, suggesting that an established immune response to *Pv*MSP1_19_ in this model can help immunity mounted later during infection.

## Introduction

Two *Plasmodium* species are responsible for the majority of malaria cases in the world: *P. falciparum* and *Plasmodium vivax*. Estimates from 2017 show that there were ~219 million cases and 435,000 deaths due to malaria (1). *P. falciparum* causes most of the severe malaria cases, with a death toll of thousands of children under the age of 5 years, mainly in Africa. *P. vivax* is present mainly in Southeast Asia and in South America (1). In Brazil, *P. vivax* is responsible for around 85% of malaria cases, affecting the population living in the Amazonian region and causing high morbidity, with an important economic impact (2).

Historically, disease eradication or efficient control has only been achieved with the use of effective vaccines. The most advanced malaria vaccine RTS,S/AS01, against *P. falciparum*, completed phase III clinical trial (3) with 27–39% efficacy after a four-dose regimen in infants and children in sub-Saharan African countries. This vaccine is based on the antigen Circumsporozoite Protein (CSP), the most abundant protein on the surface of sporozoites (4), and thus targets the pre-erythrocytic stages of the parasite. Based on the phase III trial results, RTS,S/AS01 received approval to be piloted in Malawi, Kenya, and Ghana. In parallel, efforts to improve the modest efficacy of RTS,S/AS01 observed so far include the development of vaccines targeting other stages or antigens of the parasite to be combined with the RTS,S formulation (5–8).

Malaria elimination based on vaccines will require the development of a vaccine not only against *P. falciparum*, but also against *P. vivax*. Many research groups in Brazil and abroad are working on the development of a *P. vivax* vaccine based on the *Pv*CSP antigen (9–11). However, it is reasonable to foresee, based on the results obtained with the clinical trials of RTS,S/AS01, that a *P. vivax* vaccine based only on the *Pv*CSP may not be fully effective by itself. The development of vaccines based on *P. vivax* blood stage antigens is important so they can be combined with future *Pv*CSP-based formulations. *P. vivax* blood stage antigens like the Duffy Binding Protein (*Pv*DBP), Merozoite Surface Protein 1 (*Pv*MSP1), or Apical Membrane Antigen 1 (*Pv*AMA1) continue in the pipeline of malaria vaccine development. So far, few vaccine formulations based on *P. vivax* blood stage antigens have undergone clinical trials. The lack of a continuous *P. vivax* laboratory culture has so far thwarted efficacy tests of these available vaccine formulations in pre-clinical studies, which is an obvious obstacle to progress with these formulations to clinical tests in humans. The mouse-infecting *P. berghei* has been used for efficacy tests of *Pv*CSP-based formulations (10, 11). These studies used *P. berghei* hybrid mutant lines expressing the *Pv*CSP to challenge mice immunized with formulations based on the *P. vivax* antigen. The same strategy could accelerate the pre-clinical development of formulations based on *P. vivax* blood stage antigens.

The MSP1 is the most abundant protein on the merozoite surface and therefore considered of high vaccine potential. The MSP1 high-molecular-weight precursor is synthetized during schizogony and undergoes proteolytic cleavages resulting in four polypeptides complexed on the parasite surface (12). MSP1 processing post-schizogony is essential for merozoite egress from the erythrocyte host cell (13). During merozoite invasion of a new erythrocyte host, the 42-kDa C-terminal region of MSP1, named MSP1_42_, is processed into two polypeptides, MSP1_33_ and MSP1_19_, and the bulk complex is shed from the surface (14). The 19-kDa C-terminal end, named MSP1_19_, remains attached at the merozoite surface after invasion and has been used as a protective antigen in different models (15, 16).

Many vaccine formulations based on the *Pv*MSP1_19_ or *Pv*MSP1_42_ sequences have been developed and had their immunogenicity in mice, and sometimes in non-human primates, studied (17–28). In the last years, we developed recombinant proteins based on the sequence of the *Pv*MSP1_19_ formulated in different adjuvant systems. For instance, the HIS_6_-*Pv*MSP1_19_ and HIS_6_-*Pv*MSP1_19_-PADRE recombinant proteins, the latest fused to the Pan-Allelic-DR-Epitope (PADRE), expressed in bacteria and purified, were recognized by sera from *P. vivax*-exposed individuals and showed immunogenicity in C57BL/6 and BALB/c mice (18, 19), as well as in non-human primates (21). The TLR5 agonist flagellin of *Salmonella* (FliC) was later fused to these antigens and the resulting recombinant protein HIS_6_-FliC-*Pv*MSP1_19_-PADRE was highly immunogenic in mice (24). More recently, the *Pv*MSP1_42_ sequence was fused to a recombinant mAb containing the heavy chain of the mouse αDEC205, resulting in a recombinant antibody, αDEC-*Pv*MSP1_42_, that targets the antigen directly to dendritic cells to induce high specific antibody titers (26). Although inducing strong immunological responses in different animal models, these formulations have not been tested against parasites due to the lack of *P. vivax* cultures.

Here we used the murine malaria model *P. berghei* to generate two transgenic *P. berghei* lines expressing the *Pv*MSP1_19_. These transgenic parasites were used to challenge mice immunized with vaccine formulations based on the sequences of *Pv*MSP1_19_ or *Pv*MSP1_42_.

## Experimental Procedures

### Mice

Four- to six-week-old female C57BL/6 and BALB/c mice were bred at the Isogenic Mouse Facility of the Parasitology Department, Institute of Biomedical Sciences, University of São Paulo, Brazil. All protocols were approved by the Institutional Animal Care and Use Committee (CEUA) of the Institute (protocol number 132/2014) and all the animals were handled according to the Brazilian College of Animal Experimentation guidelines. All experimental methods were performed in accordance with the National Institutes of Health Guide for the Care and Use of Laboratory Animals and the Brazilian National Law (11.794/2008).

### Plasmids

**Figure 1:**
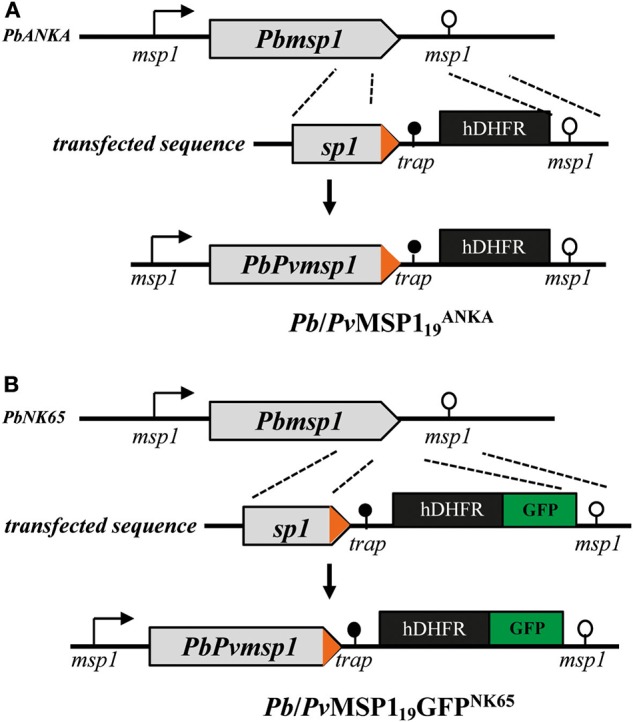
Strategy for generating the modified MSP1 locus. Modification of the MSP1 locus in *P. berghei* ANKA (*Pb*ANKA) **(A)** and in *P. berghei* NK65 (*Pb*NK65) **(B)** for the generation of the *Pb/Pv*${\text{MSP1}}_{19}^{\text{ANKA}}$ and of the *Pb/Pv*MSP1_19_GFP^NK65^ hybrid parasites, respectively. Arrow boxes, coding sequences; arrows, 5′UTR; open circle lollipop, 3′UTR of *msp1*; black circle lollipop, *trap* 3′UTR; black box, hDHFR selection marker cassette; green box, GFP cassette. *P. berghei* sequences are in gray and *P. vivax* sequences in orange.

To generate the p*Pb*/*Pv*MSP1_19_ plasmids, 1,526 bp of the sequence upstream the *P. berghei* ANKA MSP1_19_ (nucleotides 3,521–5,046 of the genomic sequence of PBANKA_0831000 in PlasmoDB.org) and the first 611 bp of the *msp1* 3′UTR were cloned flanking the sequence of the *P. vivax* MSP1_19_ (318 bp, amplified from DNA of parasites isolated from a Brazilian patient and kindly provided by Dr. Marcelo U. Ferreira) followed by the *P. berghei trap* 3′UTR (600 bp) and a human Dihydrofolate Reductase cassette (hDHFR) (29) using as background the pBlueScript (pBS-SK+) vector (Figure 1A and Supplementary Table 1 for primer sequences). The cloned *Pv*MSP1_19_ was sequenced and is identic to the sequences of *P. vivax* Sal-I and Belém strains (Supplementary Figure 1). For transfections of the *P. berghei* NK65 line, a Green Fluorescent Protein (GFP) cassette (29) was inserted between the hDHFR cassette and the *msp1* 3′UTR sequence in the p*Pb*/*Pv*MSP1_19_ plasmid at *SmaI* site (Figure 1B). The final vectors contain two homologous regions to target integration by double crossover at the *P. berghei* MSP1 locus replacing the endogenous MSP1_19_ by the *P. vivax* MSP1_19_ sequence.

### *P. berghei* Transfection and Selection

The targeting sequence was removed from the plasmid with the restriction enzymes *KpnI* and *NotI*, gel purified (QIAquick Gel Extraction Kit, ref 28706, following the manufacturer's instructions), and transfected into *P. berghei* ANKA or NK65 lines following a detailed published protocol (30). The *P. berghei* ANKA and NK65 strains differ in virulence in C57BL/6 and BALB/c mice. The ANKA strain is generally more virulent and induces experimental cerebral malaria (ECM) in C57BL/6, while the NK65 is less virulent and does not induce ECM. *P. berghei* merozoites were electroporated with 5 μg of the targeting sequence using the “U33 program” of the Nucleofector™ 2b electroporator and injected intravenously in the caudal vein of two 4-week-old female BALB/c mice. Genetically transformed parasites were selected with 0.07 mg/ml pyrimethamine (Sigma, ref 46706) in the drinking water. Pyrimethamine-resistant parasites were cloned in mice by limiting dilution.

### Genotype Analysis of Pyrimethamine-Resistant Parasites

Infected mice blood was lysed with 0.15% saponin, parasites were harvested by centrifugation for 3 min at 10,000 × g, and the pellet was washed twice with phosphate-buffered saline (PBS) and resuspended in 200 μl of PBS for gDNA purification (Qiagen “DNeasy Blood & tissue kit,” ref 69506) following the manufacturer's instructions. To confirm correct genetic modification into the target *P. berghei* locus, PCR was performed using specific primers for amplification of the WT or mutant loci (Supplementary Table 1 for primer sequences).

### Immunofluorescence Assay

Parasite blood stages were synchronized during 16 h of incubation and separated with a Nycodenz 16.5% gradient to collect mature schizonts (30), which were fixed with 4% paraformaldehyde (PFA)−0.0075% glutaraldehyde, permeabilized with 0.1% Triton X-100, and blocked with 3% BSA. After 4°C overnight incubation with either anti-mouse *Pb*MSP1 (MRA-667, *Mus musculus*, L1.6) or anti-mouse-*Pv*MSP1_19_ Mab K23 (24) diluted at 1/500 in PBS-3% BSA, the cells were washed and incubated for 30 min with Alexa Fluor® 488 conjugate [F(ab')2-Goat anti-Mouse IgG (H+L) Cross-Adsorbed Secondary Antibody, Life technologies, ref A11017], or Alexa Fluor® 568 [F(ab')2-Goat anti-Mouse IgG (H+L) Cross-Adsorbed Secondary Antibody, Life technologies, ref A11019]. Samples were then stained with DAPI and mounted in glass slides. Images were acquired on a fluorescence microscope (Axio Imager.M2, Zeiss) with a 100 × EC Plan-Neofluar 100x/1.30 Oil M27 objective (Zeiss) and processed using ImageJ-FiJi software (31).

### Western Blotting

Pelleted mature schizonts were lysed in 0.5 ml RIPA buffer containing protease inhibitor for 10 min on ice and then centrifuged. The supernatant was loaded in an SDS-PAGE under reducing conditions for separation and transferred to a nitrocellulose membrane (Hybond, Amersham, ref 10600003), which was then blocked with TBST-milk (Tris Buffered Saline Tween 20–0.05%–milk 5%). After 4°C overnight incubation with either anti-mouse *Pb*MSP1-Nt (MRA-667, *Mus musculus*, L1.6), *Pb*MSP1-Ct (kindly provided by Dr. Robert Ménard), or anti-mouse-*Pv*MSP1_19_ Mab K23 (24) diluted at 1:2,000 v:v in TBST-milk, the membranes were washed and incubated for 1 h with goat anti-mouse-HRP [Goat anti-Mouse IgG (H+L) Cross-Adsorbed Secondary Antibody, HRP, ThermoFisher, ref A16072] or goat anti-rabbit-HRP [Goat anti-Rabbit IgG (H+L) Highly Cross-Adsorbed Secondary Antibody, HRP, ThermoFisher, ref A16110] as secondary antibody at room temperature. The signal was revealed with WestFemto Supersignal (Pierce, ref 34096) and images acquired using a ChemiDoc™ Imaging System (Bio-rad).

### Immunization Regimen and Challenge

Different groups of female C57BL/6 or BALB/c (6 weeks old) mice were immunized three times subcutaneously with 10 μg of recombinant protein in combination with 50 μg Polyinosinic–polycytidylic acid [poly (I:C)] (Invivogen), at 3-week intervals. Antigens used were the following: HIS_6_-*Pv*MSP1_19_: recombinant *P. vivax* MSP1_19_ protein expressed in bacteria and purified by affinity followed by ion exchange chromatography (18); HIS_6_-*Pv*MSP1_19_-PADRE: recombinant *P. vivax* MSP1_19_ protein fused to the Pan-Allelic-DR-Epitope, PADRE, expressed in bacteria and purified by affinity followed by ion exchange chromatography (19); HIS_6_-FliC-*Pv*MSP1_19_-PADRE: recombinant *P. vivax* MSP1_19_ protein fused to the PADRE and the flagellin of *Salmonella*, expressed in bacteria and purified by affinity followed by ion exchange chromatography (24); αDEC-*Pv*MSP1_42_ recombinant mAb containing the heavy chain of the mouse αDEC205 fused to MSP1_42_ of *P. vivax* (26). The purified recombinant proteins expressed in bacteria were subjected to endotoxin removal (Pierce, ref 88274). The amino acid sequences in the vaccine antigens compared to the MSP1_19_ sequences of *P. vivax* strains and of the isolate used for the hybrid *P. berghei* strains are shown in Supplementary Figure 1. Control groups received only adjuvants or a saline solution. Three weeks after the last immunizing dose, groups were challenged intravenously with 5 × 10^3^ erythrocytes parasitized with the *P. berghei Pb*/*Pv*${\text{MSP1}}_{19}^{\text{ANKA}}$ or *P. berghei Pb*/*Pv*MSP1_19_GFP^NK65^ mutant lines, obtained from previously infected donor mice. Parasitemia was monitored daily after challenge infection by microscopic examination of stained blood smears or by flow cytometry in the case of *P. berghei Pb*/*Pv*MSP1_19_GFP^NK65^ challenge. Mice were euthanized when signs (one or more) of severe disease/illness were observed, characterized by ruffled fur, shivering, clear weight loss, irregular breathing, and difficulty to walk (32).

### Serology, Mouse *Pv*MSP_19_ Antibodies

High-binding plates (Corning, ref 3590) were coated with 50 μl of 2 μg/ml HIS_6_-*Pv*MSP1_19_ recombinant protein in PBS overnight at room temperature. The wells were blocked with a solution of PBS, 0.1% Tween (PBST) with 1% bovine serum albumin (BSA) and 5% low-fat milk for 1 h. Mice sera were added at serial three-fold dilutions starting at 1:200, v:v, and incubated for 2 h in PBST–BSA–low-fat milk. Anti-mouse IgG-HRP antibody (Peroxidase AffiniPure Goat Anti-Mouse IgG, Fcγ fragment specific, Jackson ImmunoResearch, ref 115-035-071) was added at 1:2,000, v:v, in PBST–BSA–low-fat milk in all wells and incubated for 2 h. Between all steps, plates are washed with PBS−0.02% Tween 20 (PBST). Finally, revelation buffer was added for 10 min, phosphate buffer (0.2 M, Na_2_HPO_4_), citric acid (0.2 M), pH 4.7, *o*-phenylenediamine dihydrochloride (OPD), and hydrogen peroxide (30% H_2_O_2_). Reaction was stopped with sulfuric acid (4N, H_2_SO_4_) and the optical density (OD) was read at 490 nm using an ELISA plate reader (BioTek, ELx800). Titers were determined as the log of the last serial dilution with OD >0.1. For the avidity assays, pooled sera were diluted to obtain an OD of ~1.0, and after the 2-h incubation, the wells were treated for 10 min with different concentrations of Urea ranging from 6 to 1 M in PBS. The plates were then washed for incubation with the secondary antibody and revelation.

### Merozoite Invasion Assay

*Plasmodium berghei Pb*/*Pv*MSP1_19_-GFP^NK65^ merozoites were obtained by filtering enriched cultures of schizonts, as described above for parasite preparation for transfection (30). Mature schizonts were filtered through 5-μm and then 1.2-μm filters yielding highly pure preparations of free merozoites. The merozoites were put in contact with 8 × 10^7^ mouse red blood cells in complete RPMI medium (RPMI 1640-GlutaMAX^TM^; 20% FBS; 25 mM HEPES; 25 U/L Neomycin; filter 0.22 μm) in a 96-well-flat bottom plate. For the tests, the medium contained sera from immunized mice (before challenge or on day 10 after challenge) diluted at 1:1.5, v:v. Serum from a hyper-immune mouse (repeatedly infected and cured) and 10 μM cytochalasin D (33) were used as invasion blocking controls. After 4 h of culture at 37°C, 5% CO_2_, 10% O_2_, samples were washed twice with PBS and intracellular rings were counted in blood smears.

### Cytometric Bead Array (CBA)

Groups of 4–6 weeks old female BALB/c and C57BL/6 were immunized with FliC-*Pv*MSP1_19_-PADRE or FliC-*Pv*MSP1_19_-PADRE + Poly (I:C), three times at 3-week intervals. Mice were challenged with 5 × 10^3^ *P. berghei* parasitized erythrocytes of *Pb*/*Pv*MSP1_19_-GFP^NK65^. Sera were collected at D5 before challenge and at D10 post-challenge and stored at −80°C until use. Sera were analyzed using a cytometric bead array for IL-6, IL-10, MCP-1, IFN-γ, TNF-α, and IL-12p70 proteins following manufacturer's instructions (CBA mouse inflammation kit, BD 552364). Cytokine levels were measured with FACSCalibur (BD Bioscience) and data were analyzed with FCAP Array™ Software Version 3.0 (BD Bioscience).

### Statistics and Analysis

One-way ANOVA followed by Tukey's honestly significantly different (HSD) test, or logrank (Mantel-Cox) test, were used to calculate statistical significance (*p*-values). Prism 6 software (GraphPad Software Inc., LA Jolla, CA) was used for all tests and differences were considered significant when *p* ≤ 0.05.

## Results

### Generation and Molecular Characterization of *Pb/Pv*MSP1_19_ Parasites

Wild-type (WT) *P. berghei* ANKA and *P. berghei* NK65 lines were transfected with sequences targeting integration in the MSP1 locus generating the *Pb*/*Pv*${\text{MSP1}}_{19}^{\text{ANKA}}$ and *Pb*/*Pv*MSP1_19_GFP^NK65^ mutant lines (Figures 1A,B). Replacement of the *P. berghei* MSP1_19_ by the *P. vivax* MSP1_19_ (amino acid sequence shown in Supplementary Figure 1) occurred through double crossover homologous recombination replacing the last 330 bp of the *Pbmsp1* coding sequence (PBANKA_0831000, PlasmoDB.org) by the last 318 bp of the *Pvmsp1* coding sequence amplified from purified gDNA of parasites isolated from the blood of a *P. vivax*-positive Brazilian patient. The heterologous *msp1 P. vivax* sequences are followed by the 3′ UTR of *P. berghei trap*, an hDHFR cassette for drug selection and, in the case of the NK65 line, a GFP expression cassette (Figures 1A,B). After transfection, drug selection, and cloning, PCR analysis of gDNA from the mutant parasites confirmed specific homologous recombination (not shown).

### The *Pb/Pv*${\text{MSP1}}_{19}^{\text{ANKA}}$ and *Pb/Pv*MSP1_19_GFP^NK65^ Lines Express the *P. vivax* MSP1_19_

**Figure 2:**
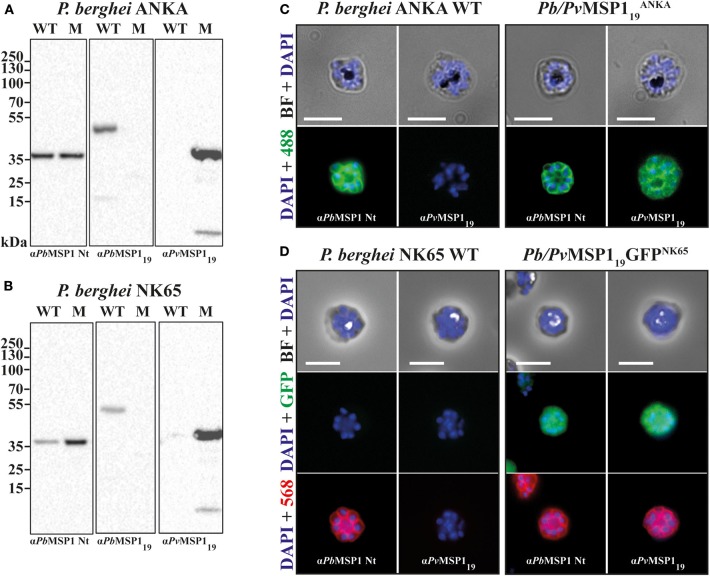
The *P. vivax* MSP1_19_ is correctly expressed in the hybrid *Pb/Pv* lines. Western blot analysis on RIPA extracts of *P. berghei* ANKA **(A)** and NK65 **(B)** wild type (WT) and mutant (M) lines schizonts using monoclonal antibodies to the N-terminal (αPbMSP1 Nt) and C-terminal (α*Pb*MSP1_19_) regions of the *P. berghei* MSP1, and to the 19-kDa C-terminal region of *P. vivax* α*Pv*MSP1_19_. Immunofluorescence on fixed and permeabilized schizonts of *P. berghei* ANKA WT and *Pb/Pv*${\text{MSP1}}_{19}^{\text{ANKA}}$ **(C)**, and of *P. berghei* NK65 WT and *Pb/Pv*MSP1_19_GFP^NK65^ **(D)** using monoclonal antibodies to the N-terminal (αPbMSP1 Nt) regions of the *P. berghei* MSP1, and to the 19-kDa C-terminal region of *P. vivax* α*Pv*MSP1_19_. Secondary antibodies are Alexa Fluor-488 or−568. Cells were mounted between lamina and diluted in PBS containing DAPI. Scale bars: 5 μm.

Correct *Pv*MSP1_19_ expression by the hybrid mutant clones was assessed by Western blot and immunofluorescence. Schizont extracts of *P. berghei* ANKA (WT), *P. berghei* NK65 (WT), *Pb*/*Pv*${\text{MSP1}}_{19}^{\text{ANKA}}$, and *Pb*/*Pv*MSP1_19_GFP^NK65^ were analyzed by Western blot with monoclonal antibodies specific to the N-terminal region of *Pb*MSP1 (α*Pb*MSP1 Nt), the C-terminal region of *Pb*MSP1 (α*Pb*MSP_19_), and α*Pv*MSP1_19_ (K23 mAb). The N-terminal region of *Pb*MSP1, not modified by the recombination events, was recognized in all extracts, while the *Pb*MSP1_19_ was only present in the WT extracts and the *Pv*MSP1_19_ in the mutant lines (Figures 2A,B). In immunofluorescence assays, the N-terminal region of *Pb*MSP1 (α*Pb*MSP1 Nt) was stained in all parasite lines showing correct expression on the merozoite surface, while α*Pv*MSP1_19_ (K23 mAb) staining was only visible on the surface of the mutant lines (Figures 2C,D). GFP expression was visible in the cytoplasm of *Pb*/*Pv*MSP1_19_GFP^NK65^ mutant merozoites (Figure 2D), enabling FACS analysis for assessment of parasitemia in infections with this line. These results confirm that the *Pb*/*Pv*${\text{MSP1}}_{19}^{\text{ANKA}}$ and *Pb*/*Pv*MSP1_19_GFP^NK65^ mutant lines correctly express *Pv*MSP1_19_ with no longer expression of *Pb*MSP1_19_.

### Intraerythrocytic Multiplication

**Figure 3:**
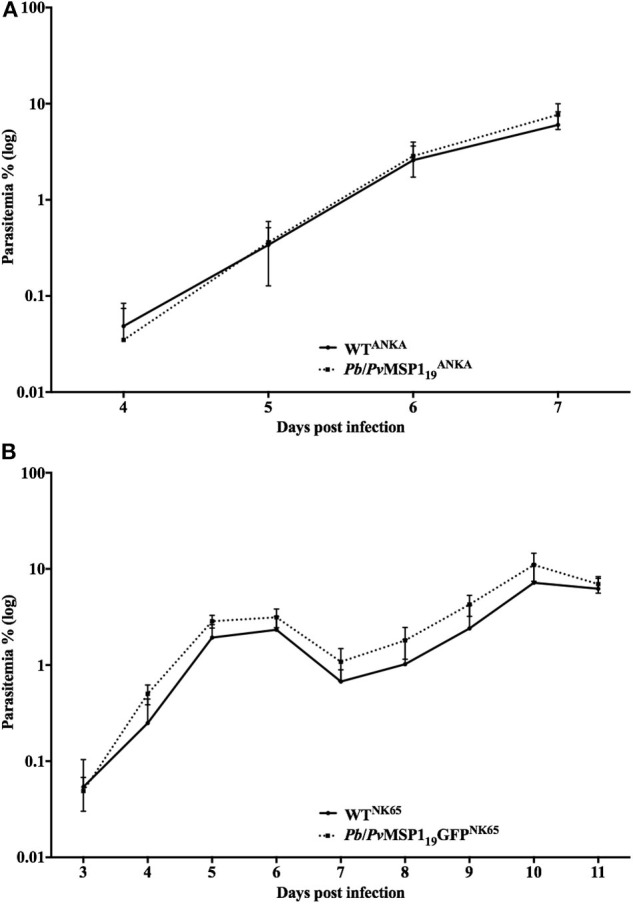
*In vivo* multiplication of the mutant *Pb/Pv*MSP1 lines is similar to that of WT parasites. **(A)** C57BL/6 mice (*n* = 5) were infected i.v. with 10^3^ red blood cells infected with *P. berghei* ANKA WT (WT^ANKA^) or with the hybrid line *Pb/Pv*${\text{MSP1}}_{19}^{\text{ANKA}}$. Parasitemia was followed daily by blood smears. **(B)** BALB/c mice (*n* = 5) were infected i.v. with 10^3^ red blood cells infected with *P. berghei* NK65 WT (WT^NK65^) or with the hybrid line *Pb/Pv*MSP1_19_GFP^NK65^. Parasitemia was followed daily by blood smears.

The asexual blood stage multiplication of the hybrid mutant lines *Pb*/*Pv*${\text{MSP1}}_{19}^{\text{ANKA}}$ and *Pb*/*Pv*MSP1_19_GFP^NK65^ was assessed by following the daily parasitemia of groups of C57BL/6 (ANKA lines) or BALB/c (NK65 lines) mice infected with 5 × 10^3^ iRBC intravenously with either the mutant lines or their respective WT background, *P. berghei* ANKA or NK65. Parasitemia was monitored for 7 days (ANKA) or 11 days (NK65) by blood smears. The intraerythrocytic development of the two mutant lines *Pb*/*Pv*${\text{MSP1}}_{19}^{\text{ANKA}}$ (Figure 3A) and *Pb*/*Pv*MSP1_19_GFP^NK65^ (Figure 3B) was similar to the WT lines. Therefore, replacement of *P. berghei* ANKA or NK65 MSP1_19_ by the *P. vivax* MSP1_19_ had no impact on the parasite ability to multiply in the host blood, indicating that host cell invasion, intraerythrocytic schizogony, and merozoite egress of the hybrid mutant merozoites occur normally.

### *Pb/Pv*${\text{MSP1}}_{19}^{\text{ANKA}}$ Challenge

**Figure 4:**
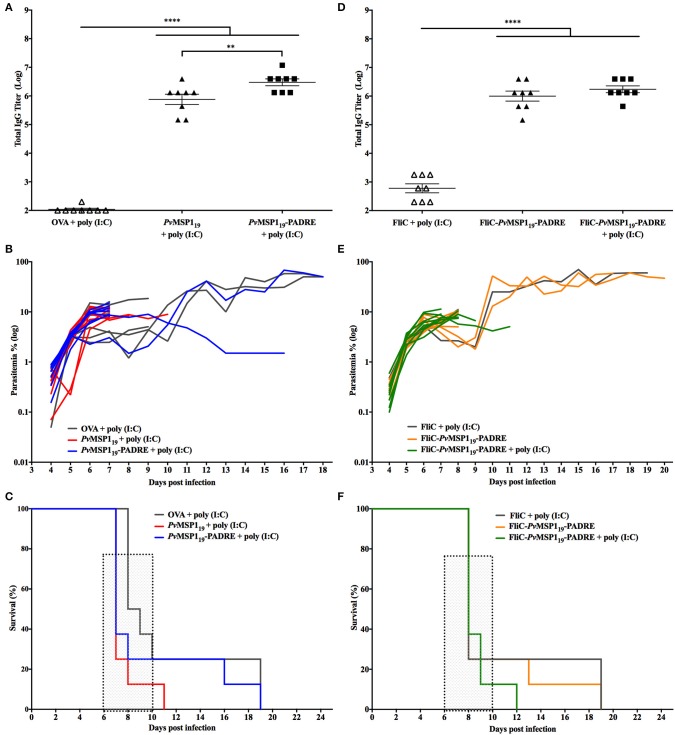
Immunizations with *Pv*MSP1_19_-based formulations do not protect C57BL/6 mice from challenge with *Pb/Pv*${\text{MSP1}}_{19}^{\text{ANKA}}$. C57BL/6 mice (*n* = 8) were vaccinated with three immunizing doses of HIS_6_-*Pv*MSP1_19_ + poly (I:C), HIS_6_-*Pv*MSP1_19_-PADRE + poly (I:C), HIS_6_-FliC-*Pv*MSP1_19_-PADRE, HIS_6_-FliC-*Pv*MSP1_19_-PADRE + poly (I:C), or with the control formulations Ovalbumin (OVA) + poly (I:C) or Flagellin (FliC) + poly (I:C). *HIS*_6_*-* is omitted from the name of the recombinant proteins in the figure. **(A,D)** Total IgG titers (Log) after the third immunizing dose. Each symbol represents one animal of the indicated groups. **(B,E)** Mice were challenged i.v. with 5 × 10^3^ red blood cells infected with the hybrid line *Pb/Pv*${\text{MSP1}}_{19}^{\text{ANKA}}$ and the parasitemia was followed daily by blood smears. Each line represents one mouse of the indicated groups. **(C,F)** Mortality curves of the indicated groups. Dashed boxes show the window of time when typic cerebral complications occur. One-way ANOVA followed by Tukey's HSD test: **p < 0.01 and ****p < 0.0001.

To test the ability of vaccine formulations based on the *Pv*MSP1_19_ to induce protective immunity in mice against the ANKA mutant line, C57BL/6 mice were immunized with HIS_6_-*Pv*MSP1_19_ + poly (I:C), HIS_6_-*Pv*MSP1_19_-PADRE + poly (I:C), HIS_6_-FliC-*Pv*MSP1_19_-PADRE, or HIS_6_-FliC-*Pv*MSP1_19_-PADRE + poly (I:C) (Figure 4). These antigens have been characterized in previous works, inducing strong specific cellular and humoral immune responses in mice (18, 21, 24). Control groups consisted of mice immunized with a non-related antigen (ovalbumin, OVA) or with purified flagellin from *Salmonella* (FliC) combined with poly (I:C). After three immunizing doses, mice that received the formulations based on the *Pv*MSP1_19_ sequence had high titers of specific anti-*Pv*MSP1_19_ in the sera compared to mice in the control groups (Figures 4A,D). Three weeks after the third immunizing dose, the groups were challenged intravenously with 5 × 10^3^ erythrocytes parasitized with the *P. berghei Pb*/*Pv*${\text{MSP1}}_{19}^{\text{ANKA}}$ line. Despite the high specific anti-*Pv*MSP1_19_ titers in the sera, mice immunized with *Pv*MSP1_19_-based formulations were not able to control parasite multiplication in the blood, as there were no differences in the time course of parasitemia in these mice compared to controls (Figures 4B,E). In all challenged mice, regardless of the immunizing group, a rapid increase of parasitemia was observed until day 6 post-challenge (Figures 4B,E) when characteristic signs of cerebral malaria, mostly ataxia and ruffled hair, started to appear. Most mice succumbed infection between days 6 and 11 post-challenge (Figures 4C,F), likely of cerebral complications. Individuals that survived after day 11 post-challenge were still not able to control parasitemia (Figures 4B,E), regardless of immunization.

### *Pb/Pv*MSP1_19_-GFP^NK65^ Challenge

**Figure 5:**
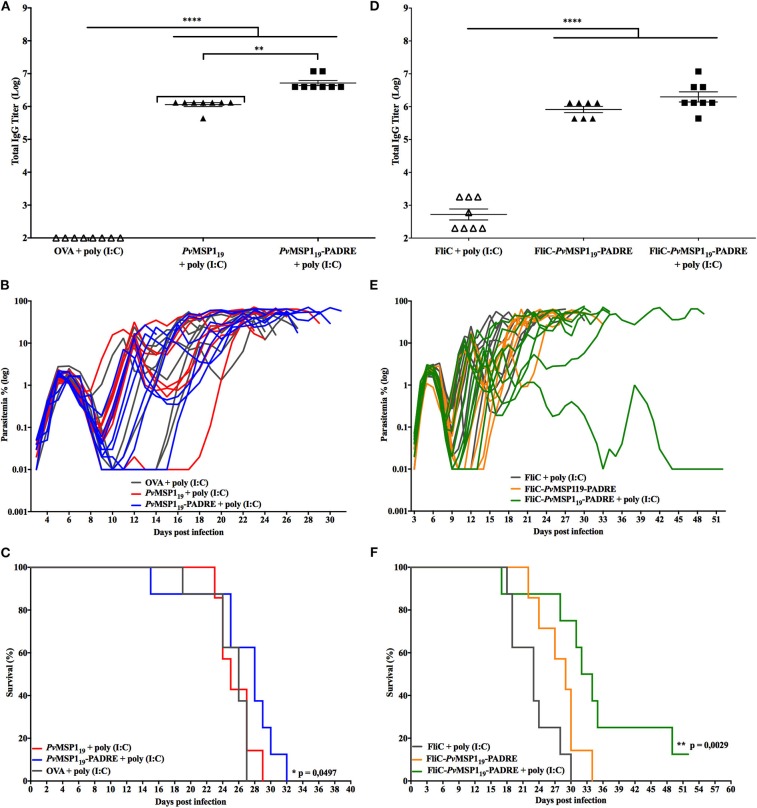
Immunizations with *Pv*MSP1_19_-based formulations partially protect C57BL/6 mice from challenge with *Pb/Pv*MSP1_19_GFP^NK65^. C57BL/6 mice (*n* = 8) were vaccinated with three immunizing doses of HIS_6_-*Pv*MSP1_19_ + poly (I:C), HIS_6_-*Pv*MSP1_19_-PADRE + poly (I:C), HIS_6_-FliC-*Pv*MSP1_19_-PADRE, HIS_6_-FliC-*Pv*MSP1_19_-PADRE + poly (I:C), or with the control formulations Ovalbumin (OVA) + poly (I:C) or Flagellin (FliC) + poly (I:C). *HIS*_6_*-* is omitted from the name of the recombinant proteins in the figure. **(A,D)** Total IgG titers (Log) after the third immunizing dose. Each symbol represents one animal of the indicated groups. **(B,E)** Mice were challenged i.v. with 5 × 10^3^ red blood cells infected with the hybrid line *Pb/Pv*MSP1_19_GFP^NK65^ and the parasitemia was followed daily by flow cytometry. Each line represents one mouse of the indicated groups. **(C,F)** Mortality curves of the indicated groups. One-way ANOVA followed by Tukey's HSD test: *p < 0.05; **p < 0.01; and ****p < 0.0001. For mortality curves: Mantel-Cox test.

Since the *P. berghei* ANKA strain is known to produce very virulent infections in C57BL/6 mice, we tested the ability of vaccine formulations based on the *Pv*MSP1_19_ to induce protective immunity in C57BL/6 mice against the *P. berghei* NK65 strain, which does not induce cerebral complications. For this, C57BL/6 mice were immunized with the same formulations described in the section above and challenged 3 weeks after the third immunizing dose with 5 × 10^3^ erythrocytes parasitized with the *P. berghei Pb*/*Pv*MSP1_19_GFP^NK65^ mutant line. Three immunizing doses with *Pv*MSP1_19_-based formulations induced high specific anti-*Pv*MSP1_19_ titers in the sera of mice when compared to mice from the control groups (Figures 5A,D). As observed with the ANKA mutant line, in all mice challenged with the NK65 mutant line, a rapid increase of parasitemia was observed until day 6 post-challenge (Figures 5B,E), indicating that the specific anti-*Pv*MSP1_19_ induced by vaccination are not able to control parasite multiplication. After day 6 post-challenge, almost all mice, regardless of the immunizing group, controlled the infection as observed by a drop in parasitemia, which increased again at day 8 post-challenge (Figures 5B,E), a characteristic course of parasitemia over time observed in mice infected with the *P. berghei* NK65 strain (Figure 3B). Mice infections with the *P. berghei* NK65 strains can last for more than 30 days and were thus followed for up to 52 days after challenge, with parasitemia monitored daily. The mice immunized with HIS_6_-*Pv*MSP1_19_ + poly (I:C) (Figure 5C) or with HIS_6_-FliC-*Pv*MSP1_19_-PADRE (Figure 5F) died between days 20 and 32 post-challenge like the mice in the control groups. In contrast, mice immunized with HIS_6_-*Pv*MSP1_19_-PADRE + poly (I:C) (Figure 5C) or HIS_6_-FliC-*Pv*MSP1_19_-PADRE + poly (I:C) (Figure 5F) survived longer after the challenge, with two mice immunized with HIS_6_-FliC-*Pv*MSP1_19_-PADRE + poly (I:C) being able to control the parasitemia, one of them clearing the parasites from the blood.

**Figure 6:**
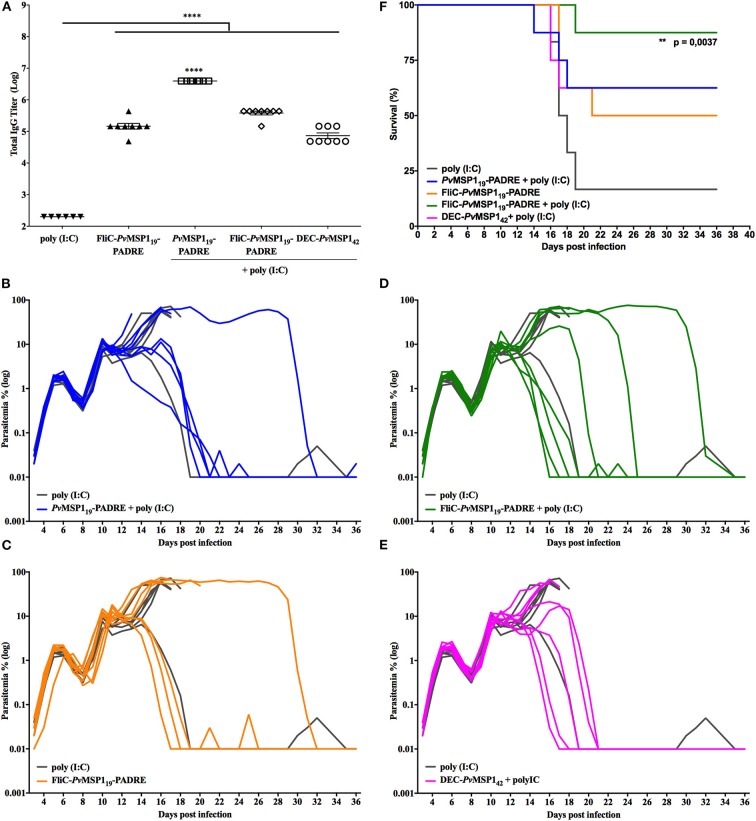
Immunizations with HIS_6_-FliC-*Pv*MSP1_19_-PADRE + poly (I:C) protects BALB/c mice from challenge with *Pb/Pv*MSP1_19_GFP^NK65^. BALB/c mice (*n* = 8) were vaccinated with three immunizing doses of HIS_6_-*Pv*MSP1_19_-PADRE + poly (I:C), HIS_6_-FliC-*Pv*MSP1_19_-PADRE, HIS_6_-FliC-*Pv*MSP1_19_-PADRE + poly (I:C), αDEC-*Pv*MSP1_42_ + poly (I:C), or poly (I:C) alone as a control (*n* = 6). *HIS*_6_*-* is omitted from the name of the recombinant proteins in the figure. **(A)** Total IgG titers (Log) after the third immunizing dose. Each symbol represents one animal of the indicated groups. **(B–E)** Mice were challenged i.v. with 5 × 10^3^ red blood cells infected with the hybrid line *Pb/Pv*MSP1_19_GFP^NK65^ and the parasitemia was followed daily by flow cytometry. Each line represents one mouse of the indicated groups. **(F)** Mortality curves of the indicated groups. One-way ANOVA followed by Tukey's HSD test: **p < 0.01 and ****p < 0.0001. For mortality curves: Mantel-Cox test.

To further investigate the ability of the *Pv*MSP1_19_-based formulations to induce protective immunity against *P. berghei* NK65 challenge, we immunized BALB/c mice, more resistant than C57BL/6 mice to *P. berghei* infections, with HIS_6_-*Pv*MSP1_19_-PADRE + poly (I:C), HIS_6_-FliC-*Pv*MSP1_19_-PADRE, HIS_6_-FliC-*Pv*MSP1_19_-PADRE + poly (I:C), or αDEC-*Pv*MSP1_42_ + poly (I:C), a recombinant mAb containing the heavy chain of the mouse αDEC205 fused to MSP1_42_ of *P. vivax* (26). All *Pv*MSP1-based formulations induced high specific antibody titers in BALB/c mice (Figure 6A). As observed after C57BL/6 mice challenged with the *P. berghei* NK65 strain (Figures 5B,E), BALB/c mice could not control initial parasitemia after challenge (Figures 6B–E). In all mice of either control or immunized groups, the parasites multiplied until day 6 post-challenge in a first wave of parasitemia, which decreased between days 6 and 9 post-challenge and came up again until day 11 in a second wave of parasitemia (Figures 6B–E). After day 11, parasitemia increased in almost all mice in the control group, which died between days 16 and 19 post-challenge (Figure 6F). On the other hand, at least half the mice immunized with *Pv*MSP1-based formulations controlled parasitemia after day 11 (Figures 6B–E) and survived infection (Figure 6F). In the group immunized with HIS_6_-FliC-*Pv*MSP1_19_-PADRE + poly (I:C), only one mouse died from infection (Figure 6D), with this formulation being the most efficient in inducing protective immunity in BALB/c mice challenged with the *P. berghei* NK65 mutant strain.

### Immunological Responses Induced by the Protective Immunization of BALB/c Mice

**Figure 7:**
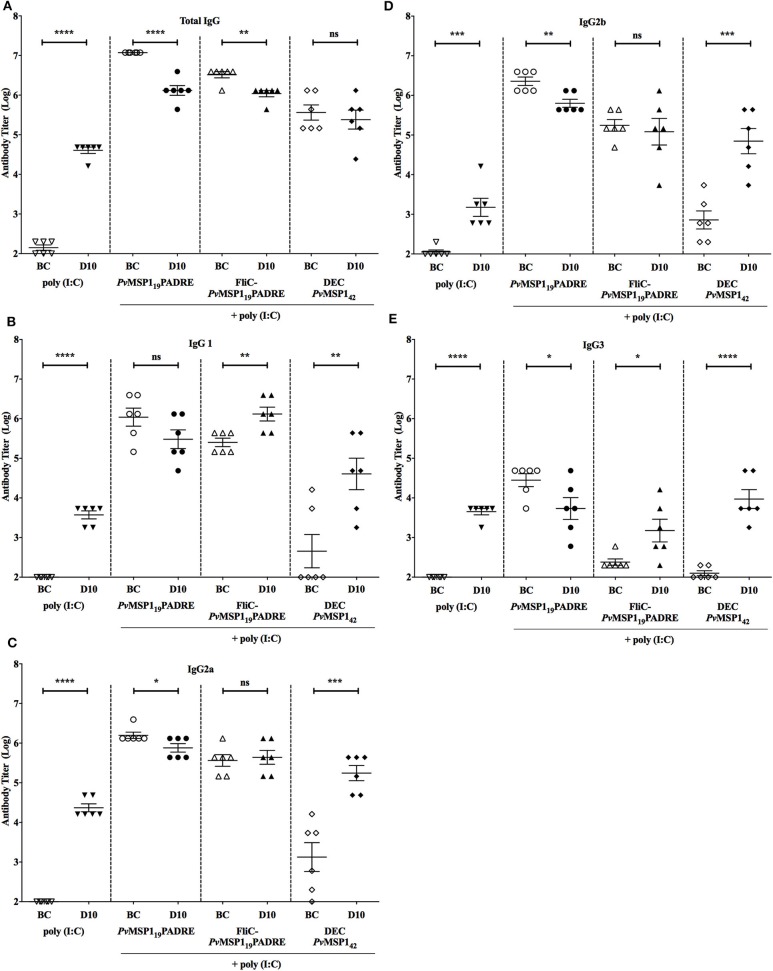
Antibody responses in vaccinated mice before challenge and at day 10 post-challenge. Sera titers of total IgG **(A)**, IgG1 **(B)**, IgG2a **(C)**, IgG2b **(D)**, and IgG3 **(E)** of BALB/c mice in each indicated group (*n* = 6) before challenge (BC) and at day 10 (D10) post-challenge. Unpaired *t*-test: ns, non-significant; *p < 0.05; **p < 0.01; ***p < 0.001; and ****p < 0.0001.

To explore which immunological mediators could be responsible for the protective immunity induced by the formulations used for vaccination of BALB/c mice, we first characterized the humoral immune response induced by vaccination immediately before challenge and on day 10 post-challenge, when parasitemia control starts in vaccinated mice. As already shown (Figure 6A), all *Pv*MSP1-based formulations induced high titers of *Pv*MSP1_19_-specific total IgG, which were maintained on day 10 post-challenge (Figure 7A). At day 10 post-challenge, an anti-parasite humoral response is already present, as observed by the increase of specific antibody titers in the control mice (Figure 7A). This increasing humoral response to parasite antigens may explain the small decrease of specific anti-*Pv*MSP1_19_ titers in the groups immunized with HIS_6_-*Pv*MSP1_19_-PADRE + poly (I:C) and HIS_6_-FliC-*Pv*MSP1_19_-PADRE + poly (I:C) (Figure 7A), likely due to an exhaustion of the immune response. The *Pv*MSP1_19_-specific titers before challenge and 10 days post-challenge of the IgG subclasses IgG1 (Figure 7B), IgG2a (Figure 7C), IgG2b (Figure 7D), and IgG3 (Figure 7E) were maintained or increased in the most protected group, HIS_6_-FliC-*Pv*MSP1_19_-PADRE + poly (I:C), and in the group that received αDEC-*Pv*MSP1_42_ + poly (I:C), while in the group immunized with HIS_6_-*Pv*MSP1_19_-PADRE + poly (I:C), the subclasses titers were reduced at day 10 compared to before challenge. These results indicate that there is an evident, yet expected, modulation of the humoral specific immune response to *Pv*MSP1_19_ in the course of infection. The relative increase in subclasses titers in the group immunized with αDEC-*Pv*MSP1_42_ + poly (I:C) is likely due to the lower titers in this group before challenge, while the maintenance or increase of the subclasses titers in the group immunized with HIS_6_-FliC-*Pv*MSP1_19_-PADRE + poly (I:C) may be linked to the better protection observed for this group.

**Figure 8:**
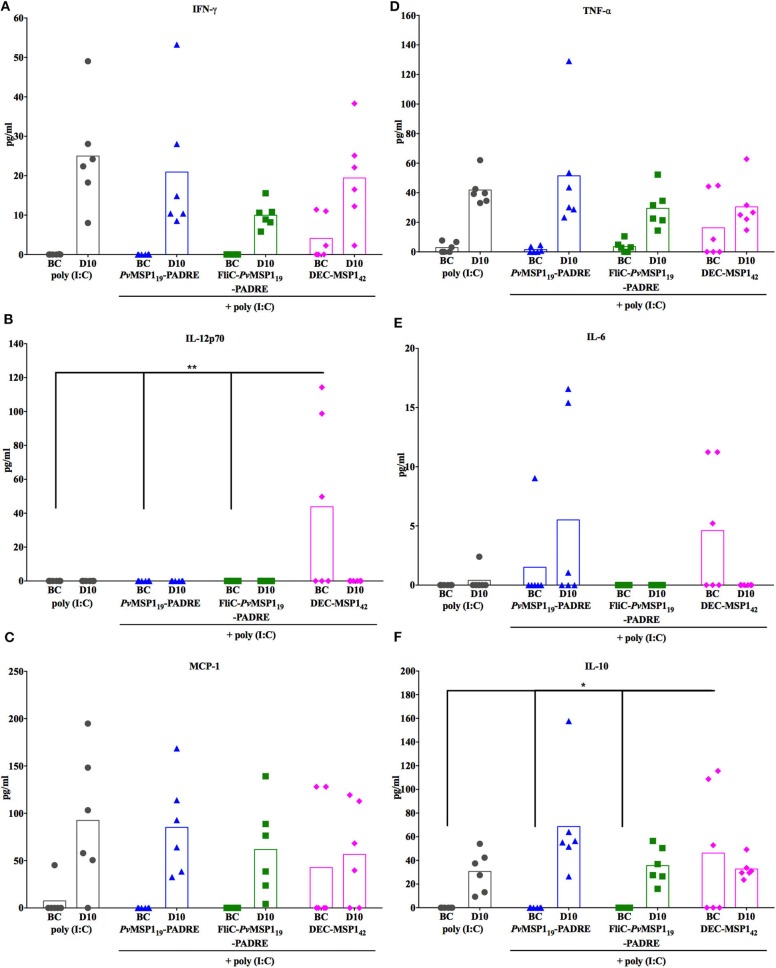
Cytokine responses in vaccinated mice before challenge and at day 10 post-challenge. The amounts of IFN-γ **(A)**, IL-12p70 **(B)**, MCP-1 **(C)**, TNF-α **(D)**, IL-6 **(E)**, and IL-10 **(F)** were measured by CBA in the sera of mice from the indicated groups (*n* = 6) before challenge (BC) and at day 10 (D10) post-challenge. One-way ANOVA followed by Tukey's HSD test: ns, non-significant; *p < 0.05 and **p < 0.01.

**Figure 9:**
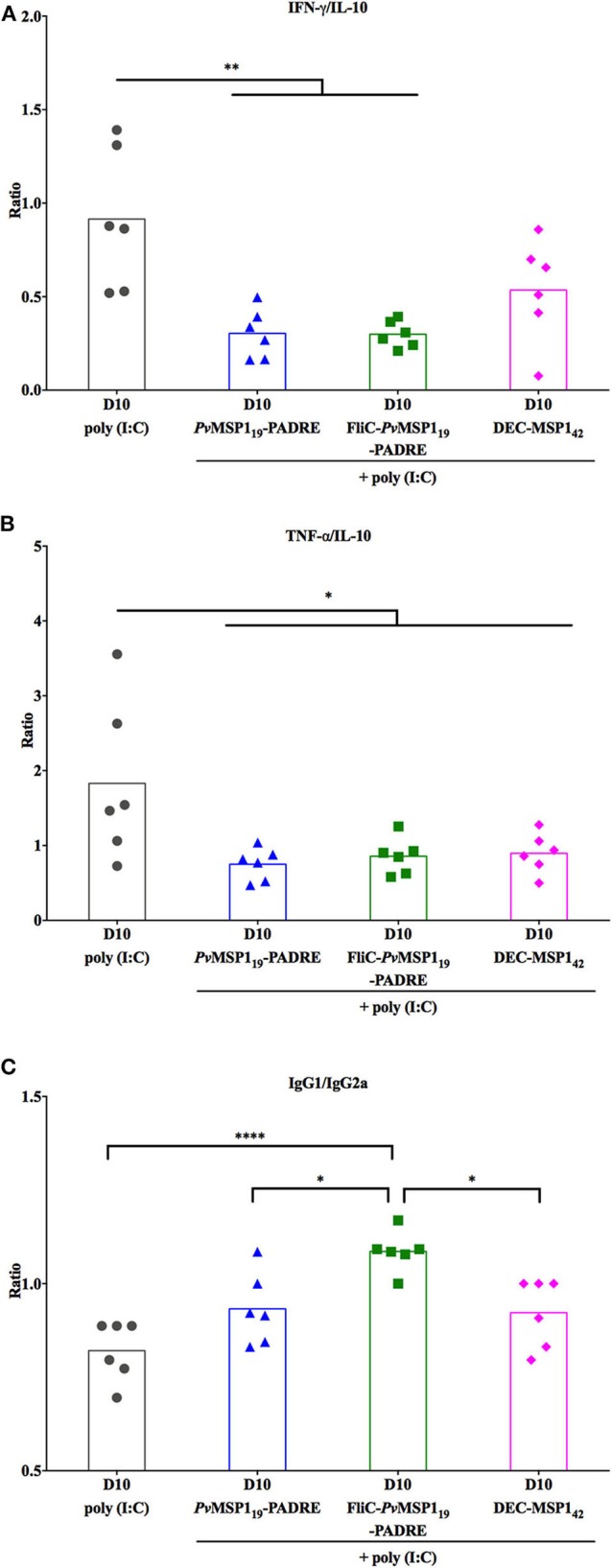
Cytokine and IgG subclasses ratios in vaccinated mice at day 10 post-challenge. IFN-γ/IL-10 **(A)**, TNF-α /IL-10 **(B)**, and IgG1/IgG2a **(C)**. One-way ANOVA followed by Tukey's HSD test: *p < 0.05; **p < 0.01; and ****p < 0.0001.

We then investigated whether the survival of the vaccinated mice without initial parasite control could be explained by a modulation of the inflammatory status during infection. For this, the levels of serum inflammatory and anti-inflammatory cytokines were measured before challenge (BC) and on day 10 post-challenge (D10) (Figure 8). An overall increase in cytokine levels due to infection was observed (BC to D10). On the other hand, there were no important differences when comparing in the same time points (BC × BC and D10 × D10 between groups) the absolute sera levels of the inflammatory cytokines interferon gamma (IFN-γ), tumor necrosis factor alpha (TNF-α), interleukin (IL) 12p70 (IL-12p70), IL-6, and monocyte chemoattractant protein 1 (MCP-1), as well as of the immunoregulatory cytokine IL-10 (Figures 8A–F). Despite no important differences in the absolute levels of the measured cytokines on D10 post-challenge, the immunized protected groups had a more balanced inflammatory systemic response as observed by the ratios IFN-γ/IL-10 (Figure 9A) and TNF-α /IL-10 (Figure 9B), also reflecting in a higher ratio IgG1/IgG2a (Figure 9C) in the most protected group immunized with HIS_6_-FliC-*Pv*MSP1_19_-PADRE + poly (I:C). These results indicate that immunization with the *Pv*MSP1_19_-based formulations promotes an immunological environment that controls the systemic inflammatory process induced by *Plasmodium* infection, which may contribute to protection by reducing tissue damage. However, a balanced inflammatory response does not explain the parasitemia control observed in the challenged protected mice after D10 post-challenge (Figures 6B–E).

### Merozoite Invasion Inhibition by Sera From Immunized Mice

**Figure 10:**
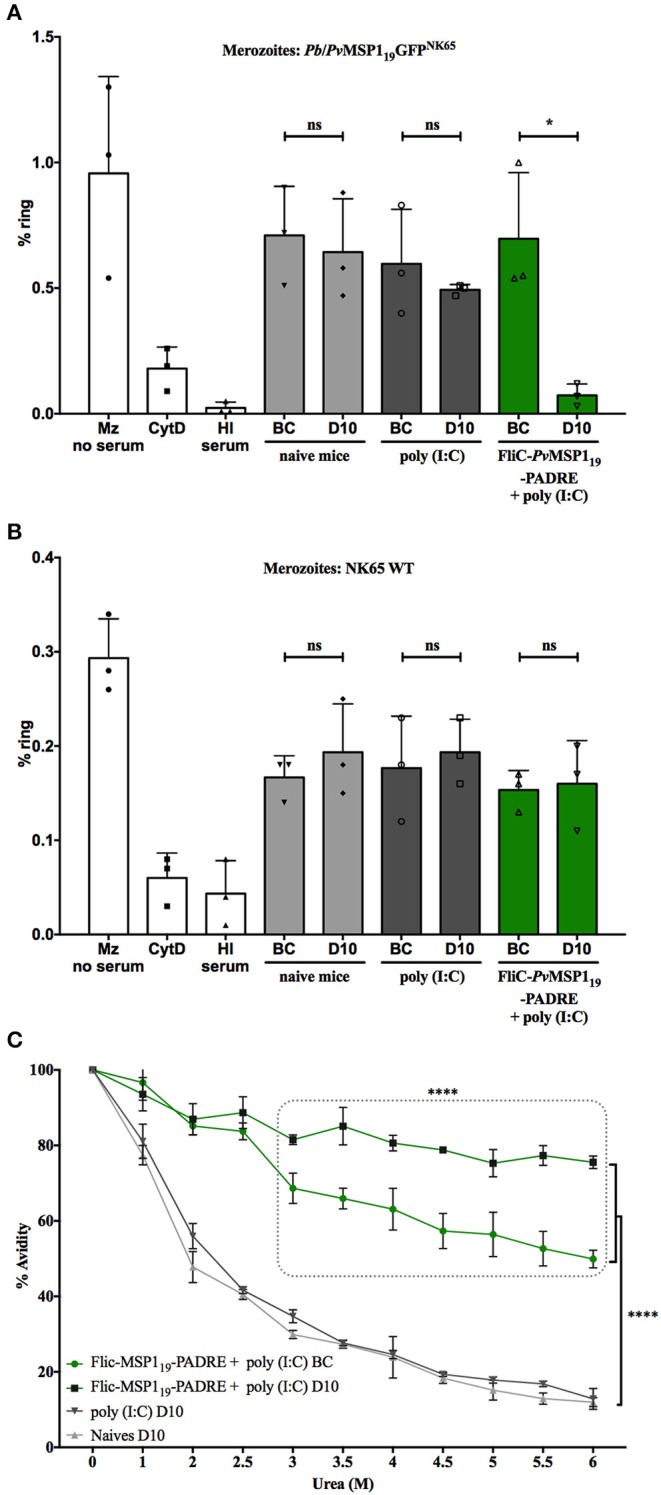
Sera from mice immunized with HIS_6_-FliC-*Pv*MSP1_19_-PADRE + poly (I:C) and challenged inhibit merozoite invasion and has increased antibody affinity to *Pv*MSP1_19_. Inhibition of *Pb/Pv*MSP1_19_GFP^NK65^ merozoites **(A)** or NK65 wild type (WT) merozoites **(B)** invasion of erythrocytes *in vitro* with pooled sera taken from naïve or poly (I:C) control mice, or from mice immunized with HIS_6_-FliC-*Pv*MSP1_19_-PADRE + poly (I:C) before challenge (BC) and at day 10 (D10) post-challenge with *Pb/Pv*MSP1_19_GFP^NK65^. Controls are assays with merozoites and erythrocytes with no added serum (Mz no serum), with cytochalasin D (CytD) or with added hyperimmune serum (HI serum). **(C)** Specific antibody affinity to *Pv*MSP1_19_ of pooled sera taken from mice immunized with HIS_6_-FliC-*Pv*MSP1_19_-PADRE + poly (I:C) before challenge (BC) and at day 10 (D10) post-challenge with *Pb/Pv*MSP1_19_GFP^NK65^, and of sera taken from naïve or poly (I:C) control mice at day 10 (D10) post-challenge with *Pb/Pv*MSP1_19_GFP^NK65^. **(A,B)** Unpaired *t*-test: ns, non-significant; *p < 0.05. **(C)** One-way ANOVA followed by Tukey's HSD test: ****p < 0.0001.

The logic of using merozoite surface antigens as vaccine targets is to induce antibodies that block merozoite invasion of host erythrocytes. Therefore, we assessed the ability of the immunized mice sera to inhibit merozoite invasion *in vitro*. For this, isolated *P. berghei Pb*/*Pv*MSP1_19_GFP^NK65^ (Figure 10A) or WT NK65 (Figure 10B) merozoites were incubated with fresh mouse erythrocytes for 4 h and the number of infected cells (with early trophozoites—rings) was counted. As a positive control of invasion, the assay was done without added mouse serum. Negative controls of invasion were done by adding cytochalasin D (CytD) to the assay to block merozoite invasion or serum from a hyperimmune mouse (HI). Sera collected from naïve or immunized mice before challenge (BC) or on day 10 post-challenge (D10) with the *Pb*/*Pv*MSP1_19_GFP^NK65^ were tested. Except for the HI serum, mice sera collected before challenge had no invasion inhibitory activity against mutant (Figure 10A) or WT (Figure 10B) merozoites regardless of immunization. This concurs with the observations that immunization of mice with the *Pv*MSP1_19_-based formulations did not induce an immunological response able to control initial parasitemia after infection (Figures 4–6). On the other hand, while 10 days of infection was not able to induce merozoite invasion inhibition by sera from naïve or poly (I:C) control mice (Figure 10), sera collected on day 10 post-challenge of mice immunized with HIS_6_-FliC-*Pv*MSP1_19_-PADRE + poly (I:C) inhibited invasion of erythrocytes by *Pb*/*Pv*MSP1_19_GFP^NK65^ merozoites (Figure 10A) and not by WT NK65 merozoites (Figure 10B). These results reveal that specific humoral responses to *Pv*MSP1_19_ raised by immunization may play a role in the control of parasitemia observed in vaccinated mice after day 10 post-challenge. We then tested the avidity of the specific total IgG antibodies to *Pv*MSP1_19_ induced by vaccination with HIS_6_-FliC-*Pv*MSP1_19_-PADRE + poly (I:C) before and after challenge compared with the avidity of the antibodies induced during the course of infection in control mice. Although they were not able to mediate control of parasite growth in the first days of infection (Figure 6D), the antibodies induced by vaccination with HIS_6_-FliC-*Pv*MSP1_19_-PADRE + poly (I:C) have higher avidity to *Pv*MSP1_19_ than the antibodies that are raised during the infection of control mice (Figure 10C). Interestingly, the avidity of the specific antibodies in vaccinated mice increases during infection, and at day 10 post-challenge, their binding to the antigen is significantly stronger than before challenge (Figure 10C). This increment in avidity to *Pv*MSP1_19_ may be necessary for the invasion-inhibitory activity observed at this time point and crucial for the protection of the immunized mice.

## Discussion

We have generated two new lines of *P. berghei* expressing the *P. vivax* MSP1_19_ in place of the endogenous *P. berghei* MSP1_19_. As previously observed with a *P. berghei* line expressing the *P. falciparum* MSP1_19_ (34), the hybrid *Pb/Pv* lines generated here expressed the heterologous antigen correctly on the merozoite surface without impacting the parasite asexual growth. Immunizations with recombinant proteins based on the sequence of the *Pv*MSP1_19_ were not able to protect C57BL/6 mice from challenge with the *P. berghei* ANKA line expressing *Pv*MSP1_19_, likely due to the virulence of these infections leading to neurological complications in this mouse strain. On the other hand, *Pv*MSP1_19_-based vaccines protected C57BL/6 and specially BALB/c mice from challenge with the *P. berghei* NK65 line expressing *Pv*MSP1_19_. More than 50% of the vaccinated BALB/c mice survived the challenge with the NK65 line, a protection that was not mediated by initial parasite control, but by modulation of systemic inflammation and late parasite control.

The murine malaria model *P. berghei* has been explored for assessing the utility of *P. falciparum* MSP1 as a protective antigen. The levels of *Pf* MSP1_19_-specific invasion inhibitory activity *in vitro* of sera from semi-immune BALB/c mice correlated with parasitemia control in the same mice after challenge with a *P. berghei* ANKA line expressing *Pf* MSP1_19_ (*Pb/Pf* M19) (34). The *Pb/Pf* M19 chimeric strain was also used to challenge BALB/c mice receiving passive transfer of rabbit anti-*Pf* MSP1_42_ IgG, with complete sterile protection observed (35). In two other studies, passive transfer of rabbit anti*-Pf* MSP1_19_ or immunizations with formulations based on the *Pf* MSP1_19_ sequence did not protect BALB/c or C57BL/6 mice from challenge with *Pb/Pf* M19 (36, 37). These disparities may be due to the use of different antigens and formulations for the mouse vaccinations and for raising rabbit antibodies for passive transfers, as well as for differences in mice colonies that may alter the kinetics of mouse infections with *P. berghei*. In our hands, no immunization with *Pv*MSP1_19_-based vaccines afforded initial control of parasite growth in mice challenged with the *Pb/Pv* chimeric lines, concurring with the absence of protection observed in these latter studies. However, we observed parasite control and protection after day 10 post-challenge of vaccinated mice, which may relate to the invasion inhibitory activity against *Pb/Pf* M19 of sera from semi-immune BALB/c mice previously observed (34). Interestingly, in a work using another murine malaria model of *P. chabaudi* infection of NMRI mice, vaccination with membranes of infected erythrocytes conferred protection against challenge without initial parasitemia control (38).

The immunological mechanisms that mediate late control of parasitemia after immunization are not fully clear. Our assays of *in vitro* merozoite invasion of erythrocytes showed that sera from BALB/c mice immunized with a protective formulation had no invasion inhibition activity before challenge, while almost completely inhibiting merozoite invasion when taken from the infected mice on day 10 post-challenge. This invasion inhibition was dependent on specific anti-*Pv*MSP1_19_, since the same sera could not inhibit *P. berghei* NK65 WT merozoite invasion of erythrocytes. The antibodies to *Pv*MSP1_19_ raised through vaccination appear to gain invasion–inhibitory activity during the course of infection. This increment in inhibitory activity is accompanied by an increase in antibody avidity to the antigen. The presence of the antigen during infection likely induces B cell affinity maturation in the germinal centers, and after 10 days of challenge, the secretion of antibodies with higher affinity starts to mediate parasite control. The gain in inhibitory activity may also be related to the recent observation that non-inhibitory monoclonal antibodies (mAbs) to the *P. falciparum* Reticulocyte binding protein homolog 5 (PfRh5) reduces the speed of merozoite invasion, increasing the time merozoites are target to invasion blocking antibodies and consequently increasing the invasion inhibition activity of inhibitory mAbs to PfRh5 (39). It is possible that, likewise, the presence of high titers of specific anti-*Pv*MSP1_19_ in vaccinated mice facilitate the invasion blocking activity of antibodies to other parasite antigens that are raised in the course of infection.

While the protective immunity induced after challenge involved the appearance of better antibodies to *Pv*MSP1_19_, it was only seen with the NK65 strain challenges. It has been proposed that *P. berghei* NK65 infections involve greater numbers of latent circulating merozoites than ANKA infections (40). The presence of more latent merozoites in NK65 infections would contribute to greater antigen exposure, increasing the effect of natural boost in infections with this strain in the vaccinated mice.

In our model, a part of the protective immunity observed after day 10 post-challenge can also be attributed to a modulation of the systemic inflammatory status of the infected mice. Mice immunized with *Pv*MSP1_19_-based formulations had lower IFN-γ/IL-10 and TNF-α/IL-10 ratios 10 days post-challenge, indicating a more balanced inflammatory response in these mice apparently mediated by reduced productions of IFN-γ and TNF-α rather than upregulation of IL-10. This immunomodulation may reduce immunopathology. While it is clear that proinflammatory immune responses are important to mediate parasite clearance in rodent infections and in humans (41), excessive production of inflammatory cytokines like IFN-γ and TNF-α is linked to the pathology associated with blood stage rodent infections (42). In humans, it is also clear that *P. falciparum* and *P. vivax* malaria pathology is largely mediated by proinflammatory cytokines like IFN-γ and TNF-α and higher levels of these cytokines or lower levels of IL-10 associate with severe diseases (43).

The most protective formulation tested in our work includes two adjuvants, poly (I:C) and flagellin, the latter being fused to the antigen. Formulations using only poly (I:C) as the adjuvant, in which the antigen was not fused to flagellin, conferred lower protection levels. Poly (I:C) is a TLR3 agonist (44), and flagellin is a potent adjuvant able to induce TLR5 (45) as well as NLRC4 (46) and NAIP5 (47) activation. The effects of flagellin in the cellular and humoral immune responses have been described in different models *in vitro* and *in vivo* (48, 49), inducing strong antibody responses with production of different IgG subtypes (including IgG1 and IgG2a) protective against a variety of pathogens (49). The strong immunomodulatory effects of flagellin combined with poly (I:C) may explain the protection immunity conferred by HIS_6_-FliC-*Pv*MSP1_19_-PADRE + poly (I:C) immunizations.

Only few human trials assessing efficacy have been performed with formulations based on the sequence of the *P. falciparum* MSP1_42_ (50, 51) with discouraging results. Although, in a pilot study, a delay in the time to diagnosis was observed in volunteers vaccinated with *Pf* MSP1_42_ and challenged with sporozoites, this was not confirmed in an expanded trial (51). In a Phase IIb clinical trial conducted in Kenya, vaccination with *Pf* MSP1_42_ did not delay the time for first malaria clinical episode in young children (50). These studies used as primary outcome time for patent parasitemia or first clinical malaria episode. Our results indicate that protective effects of MSP1_19_- or MSP1_42_-based vaccine formulations may be present only later in the course of infection, implying that clinical trials using these antigens would need to take the challenging and possibly unethical task of having outcomes on disease severity. *P. vivax* MSP1_19_ are still in pre-clinical evaluation (52), with many formulations being developed, like the ones we used in this work. Since *P. vivax* infections are less severe, it may be possible for controlled trials to have later outcomes assessing vaccination effect during infection and not aiming at sterile protection (patent parasitemia). Vaccine-induced sterile immunity to *Plasmodium* asexual stages seems indeed an almost impossible challenge, since naturally acquired immunity is not sterile and diminishes without constant exposure to infectious bites (53), but a vaccine targeting these stages and inducing clinical immunity may greatly reduce the tolls on development in early infancy imposed by *P. vivax* endemicity (54).

## Data Availability Statement

The datasets generated for this study are available on request to the corresponding author.

## Ethics Statement

This animal study was reviewed and approved by the Committee on the Ethics of Animal Experiments of the Institute of Biomedical Sciences of University of São Paulo, Brazil (CEUA No. 132/2014).

## Author Contributions

ID and DB designed the study and wrote the manuscript. ID, TC, AG, OM, and KA performed research work. ID, SB, IS, and DB analyzed the data. CM, SB, and IS contributed reagents and materials. All authors read and approved the final version of the manuscript.

### Conflict of Interest

The authors declare that the research was conducted in the absence of any commercial or financial relationships that could be construed as a potential conflict of interest.
