# Supplementary material for: Protective Immunity in Mice Immunized With P. vivax MSP119-Based Formulations and Challenged With P. berghei Expressing PvMSP119
Source: Front Immunol. 2020 Feb 19;11:28. doi: 10.3389/fimmu.2020.00028 (PMC7045055; doi:10.3389/fimmu.2020.00028)
Supplement: Supplementary file 1 [file Data_Sheet_1.pdf]

Supplementary Material

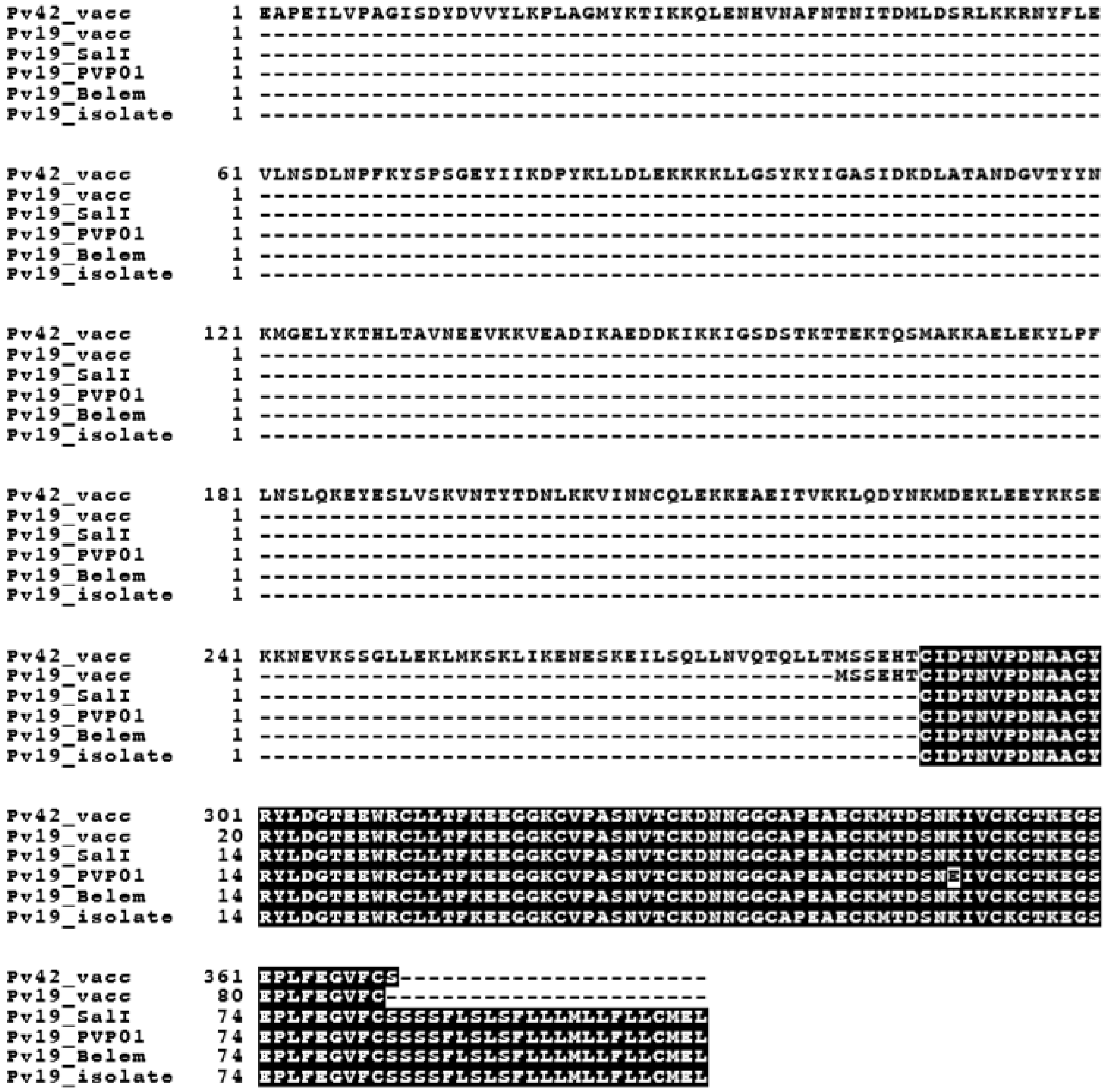

Supplementary Figure 1. Alignement of the *Pv*MSP1 amino acid sequences present in the αDEC-*Pv*MSP1$_{42}$ (Pv42_vacc) and all the MSP1$_{19}$ formulations (Pv19_vacc), compared to the *Pv*MSP1$_{19}$ amino acid sequences of the *P. vivax* strains SalI (PlasmoDB: PVX_099980), PVP01 (PlasmoDB: PVP01_0728900) and Belém (GenBank: AAN86208.1), as well as with the sequence obtained from a field isolate and used to generate the *P. berghei* hybrid parasites.

Supplementary Table 1.

**Target**	**Primer name**	**Sequence 5'- 3'**
Plasmid constructs		
hDHFR	hDhfr_Fw_EcoRI	GGG**GAATTC**TGCAGCCCAGCTTAATTCTTTTC
hDHFR	hDhfr_Rv_SmaI	AGT**CCCGGG**AAATTGAAGGAAAAAACATCATTTGTG
P. berghei MSP1	Pb_Fw_KpnI	GGG**GGTACC**GCAATCGACAAATTAGAGAACTTAGTAT
P. berghei MSP1	Pb_Rv	TACATGCTTAGGGTCTATACCTAATAAATC
msp1 3'UTR	PbM3'_Fw_SmaI	ATG**CCCGGG**ATAAATTATTGAAATATTTGTTGGATT
msp1 3'UTR	PbM3'_Rv_NotI	GAT**GCGGCCGC**CAACAATATTTCCCCAAGCTTGTTAT
P. vivax $MSP1_{19}$	Pv_Fw	GGTATAGACCCTAAGCATGTATGTATAGACACCAATGTGCCTGAT
P. vivax $MSP1_{19}$	Pv_Rv_XhoI	GGG**CTCGAG**TTAAAGCTCCATGCACAGGAGGAAAAG
trap 3'UTR	trap3'_Fw_XhoI	GGG**CTCGAG**TTTTAATAAACATATATATCTAGATG
trap 3'UTR	trap3'_Rv_EcoRI	GGG**GAATTC**TTTTTGTTTCGTAAATTTTAAAATAAT
GFP cassette	Fw_GFP	AGT**CCCGGG**GGAAAATATACGTAATA
GFP cassette	Rv_GFP	ACT**CCCGGG**AAAATACCAATAATACC
Genotypic analysis		
MSP1	P1	GTGAAGAAGATATTCCTGCC
MSP1	P2	TCAAAGAGTGGCTCAGAACC

MSP1	P3	GCAGTTGGATCACATCCACC

Restriction sites are highlighted in bold letters
